# Interaction of galectin-3 with MUC1 on cell surface promotes EGFR dimerization and activation in human epithelial cancer cells

**DOI:** 10.1038/cdd.2017.119

**Published:** 2017-07-21

**Authors:** Tushar Piyush, Anisha R Chacko, Paulina Sindrewicz, John Hilkens, Jonathan M Rhodes, Lu-Gang Yu

**Affiliations:** 1 Gastroenterology Unit, Department of Cellular and Molecular Physiology, Institute of Translational Medicine, University of Liverpool, Liverpool L69 3GE, UK; 2 Division of Molecular Genetics, The Netherlands Cancer Institute, Amsterdam, Amsterdam 1066 CX, The Netherlands

## Abstract

Epidermal growth factor receptor (EGFR) is an important regulator of epithelial cell growth and survival in normal and cancerous tissues and is a principal therapeutic target for cancer treatment. EGFR is associated in epithelial cells with the heavily glycosylated transmembrane mucin protein MUC1, a natural ligand of galectin-3 that is overexpressed in cancer. This study reveals that the expression of cell surface MUC1 is a critical enhancer of EGF-induced EGFR activation in human breast and colon cancer cells. Both the MUC1 extracellular and intracellular domains are involved in EGFR activation but the predominant influence comes from its extracellular domain. Binding of galectin-3 to the MUC1 extracellular domain induces MUC1 cell surface polarization and increases MUC1–EGFR association. This leads to a rapid increase of EGFR homo-/hetero-dimerization and subsequently increased, and also prolonged, EGFR activation and signalling. This effect requires both the galectin-3 C-terminal carbohydrate recognition domain and its N-terminal ligand multi-merization domain. Thus, interaction of galectin-3 with MUC1 on cell surface promotes EGFR dimerization and activation in epithelial cancer cells. As MUC1 and galectin-3 are both commonly overexpressed in most types of epithelial cancers, their interaction and impact on EGFR activation likely makes important contribution to EGFR-associated tumorigenesis and cancer progression and may also influence the effectiveness of EGFR-targeted cancer therapy.

MUC1 is a large, heavily glycosylated transmembrane mucin protein expressed on the apical membrane of all normal epithelial cells. MUC1 consists of a large extracellular domain, a transmembrane region and a short cytoplasmic domain/tail. The MUC1 extracellular domain contains various numbers of tandem repeats (VNTR) that are heavily glycosylated (up to 50% of the MUC1 molecular weight) with complex *O*-linked mucin type glycans.^1^ The MUC1 cytoplasmic tail harbours several phosphorylation sites and interacts with various intracellular signalling proteins.^2, 3, 4^ In epithelial cancer cells, MUC1 is overexpressed up to 10-fold and loses its apical polarization, becoming expressed over the entire cell surface.^5, 6^ In epithelial cancer cells, MUC1 *O*-glycosylation is altered with increased expression of shorter glycans such as the oncofetal oligosaccharides GalNAc*α*- (Tn antigen), sialylated-GalNAc*α*- (sialyl-Tn antigen) and Gal*β*1,3GalNAc*α*- (Thomsen-Friedenreich, T or TF antigen).^7^ MUC1 overexpression, its loss of apical polarization and increased expression of the oncofetal carbohydrate antigens have all, individually or in combination, been associated with cancer metastasis and poor prognosis.^8^ Immunological targeting of cancer-associated MUC1 is therefore under investigation as a strategy for cancer treatment.^9^

MUC1 interacts with various cellular proteins, through both its intracellular^10^ and extracellular domains,^11^ and influences proliferation, adhesion and immunomodulation.^2, 4, 12, 13^ One such interaction is with the epidermal growth factor receptor (EGFR).^14, 15, 16^

EGFR is a member of the ErbB family of receptor tyrosine kinases that includes EGFR/ ErbB1 (Her1), ErbB2 (Her2/c-Neu), ErbB3 (Her3) and ErbB4 (Her4).^17^ EGFR is involved in the regulation of multiple cellular process including proliferation and survival and its activity is directly linked with tumorigenesis and metastasis.^17^ EGFR exists normally in an inactive conformation. Binding to its extracellular domain by ligands such as EGF induces EGFR conformation change resulting in interaction with other ErbB family proteins to form homo- or hetero-dimers^17^ and subsequent activation of EGFR tyrosine kinase and auto-phosphorylation of specific cytoplasmic domain tyrosine residues. These phosphorylated residues serve as binding sites for proteins containing Src homology and phosphotyrosine binding domains, leading to activation of downstream signalling pathways such as the Ras/extracellular signal regulated kinase (ERK) pathway, the phosphatidylinositol 3-kinase (PI3) pathway, the Janus kinase/Signal transducer and activator of transcription (JAK/ STAT) pathway,^17^ crucial in cell proliferation, migration and survival.

In physiological conditions, EGFR activation is tightly regulated by its expression and by the availability of binding ligands to ensure that cell proliferation matches tissue requirement for homeostasis. In neoplasia, however, EGFR activation is often increased due to either increased EGFR expression, EGFR mutation or increased availability of the EGFR ligand produced by the same or surrounding cells.^18, 19^ Aberrant expression of EGFR by tumours typically confers a more aggressive phenotype.^20, 21, 22^ EGFR is therefore a principal target for therapeutic intervention in cancer.

Galectin-3 is a *β*-galactoside-binding protein expressed by many types of human cells and particularly by epithelial and immune cells. Galectin-3 is distributed in the cytoplasm, nuclei, cell surface, extracellular space and in circulation. Overexpression of galectin-3 commonly occurs in cancers such as colorectal, breast, lung, prostate, pancreatic, head and neck cancer and melanoma.^23^ This overexpression can be marked (up to 30-fold) particularly in those with metastasis^24^ and is increasingly shown to influence cancer cell-cell and cancer-microenvironment communication by interaction with various galactose-terminated glycans on the cell surface as well as in the extracellular matrix.^25^

Recently studies by us and others have revealed that galectin-3 is a natural ligand of MUC1 in epithelial cancer cells.^11^ The interaction between galectin-3 and MUC1, via binding of galectin-3 to the oncofetal TF antigen on MUC1,^11^ induces MUC1 cell surface polarization and the exposure of underlying smaller cell surface molecules. This leads to increased cancer cell homotypic aggregation^26^ and cancer cell heterotypic cell adhesion to vascular endothelium,^27^ two important steps in metastasis. As MUC1 is also associated with EGFR in epithelial cancer cells, the effect of galectin-3-MUC1 interaction on MUC1 cell surface localization led us to examine the impact of their interaction on EGFR activity in epithelial cancer cells.

We show here that both the MUC1 extracellular and intracellular domains contribute to EGF-induced EGFR activation in human colon and breast cancer cells with the predominate contribution from the MUC1 extracellular domain. Binding of galectin-3 to the MUC1 extracellular domain induces MUC1 cell surface polarization and increases MUC1–EGFR interaction, leading to increased EGFR homo-/hetero-dimerization and activation.

## Results

### MUC1 extra- and intra-cellular domains both contribute to EGFR activation

Interaction between MUC1 and EGFR influences EGFR activity in breast cancer,^28^ endometrial cancer^29^ and non-small cell lung cancer^30^ cells. In this study we first tested the influence of MUC1 expression on EGFR activation in human breast epithelial and colon cancer cells and assessed the influence of MUC1 intra- and extra-cellular domains on this effect.

**Figure 1:**
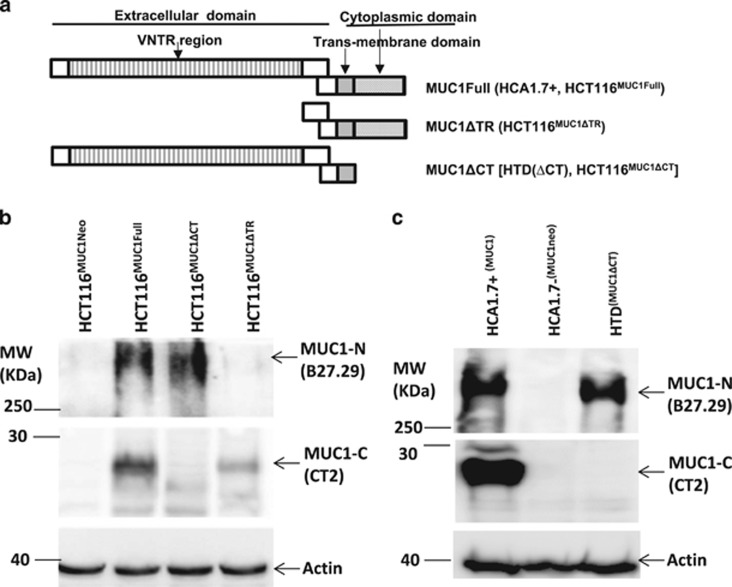
Generation of MUC1-expressing and mutant cells. (**a**) Schematic diagram of MUC1 transfectants. MUC1 expression in the transfectants of human colon cancer HCT116 (**b**), the human breast epithelial HBL-100 (**c**) cells were analysed by immunoblotting with anti-MUC1 antibodies against the MUC1 extracellular domain (B27.27) and intracellular domain (CT2). The blots were also probed with anti-actin antibody for protein loading

We transfected human colon cancer HCT116 cells with cDNA coding for full-length MUC1, MUC1 with intra- or extra-cellular domain (VNTR region) depletion (Figure 1a) and generated full-length MUC1 transfectants (HCT116^MUC1Full^), MUC1 intracellular domain-depleted (HCT116^MUC1ΔCT^) and MUC1 extracellular tandem repeat domain depleted mutants (HCT116^MUC1ΔTR^) (Figure 1b). We also obtained full-length MUC1 transfectants (HCA1.7+), negative MUC1 revertants (HCA1.7−) and MUC1 intracellular domain-depleted mutants (HTD ΔCT) from human breast epithelial HBL-100 cells (Figure 1c).

**Figure 2:**
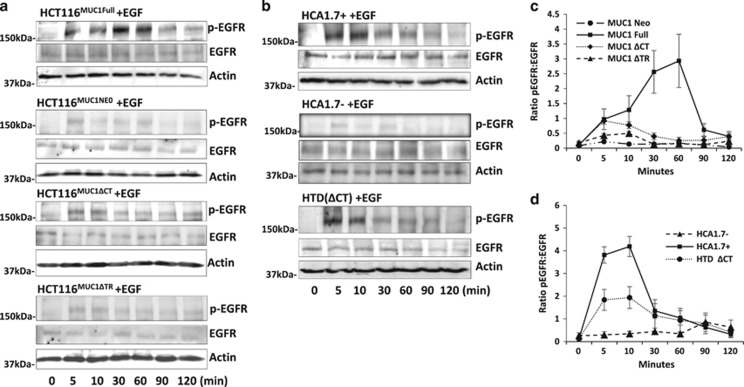
Both MUC1 extra- and intra-cellular domains influence EGF-induced EGFR activation. MUC1 transfectants of human colon (**a**) and breast (**b**) epithelial cells were treated with 20 ng/ml EGF for various times before EGFR phosphorylation were analysed by immunoblotting. The blots were also probed with anti-actin antibody for protein loading. Densitometry scanning of the bands from three independent experiments is shown in (**c**,**d**) and is expressed as ratio p-EGFR/EGF (mean±S.E.M.). The cells transfected with full-length MUC1 showed rapid EGFR phosphorylation while the MUC1-negative cells showed little response. Depletion of the MUC1 extracellular domain largely reduced, while depletion of the MUC1 intracellular domain moderately reduced, EGFR phosphorylation in comparison to the cells express full-length MUC1. Representative blots are shown in **a** and **b**

We used these transfectants to assess the effects of MUC1 on EGFR activity. In response to EGF, EGFR phosphorylation rapidly occurred in the full-length MUC1-expressing cells (HCT116^MUC1Full^ and HCA1.7+) of both cell types (Figures 2a and b) but with very weak EGFR phosphorylation in the MUC1-negative cells (Figures 2a and b). In comparison to MUC1-negative cells, a 12- and 17-fold increase of EGFR phosphorylation were observed at 10 and 60 min, respectively, of HCA1.7+ and HCT116^MUC1Full^ (Figures 2a–d). Depletion of the MUC1 extracellular domain markedly reduced EGFR activation in HCT116^MUC1ΔTR^ (Figures 2a and c) but depletion of the MUC1 intracellular domain resulted in less but still substantial reduction of EGFR phosphorylation in HTD(ΔCT) and HCT116^MUC1ΔCT^. These results suggest that expression of MUC1 is critical to EGF-induced EGFR activation and that both the MUC1 intra- and extra-cellular domains contribute to the MUC1-associated increase of EGFR activity but with predominate influence from the MUC1 extracellular domain.

### Galectin-3 interaction with cell surface MUC1 promotes EGFR activation

**Figure 3:**
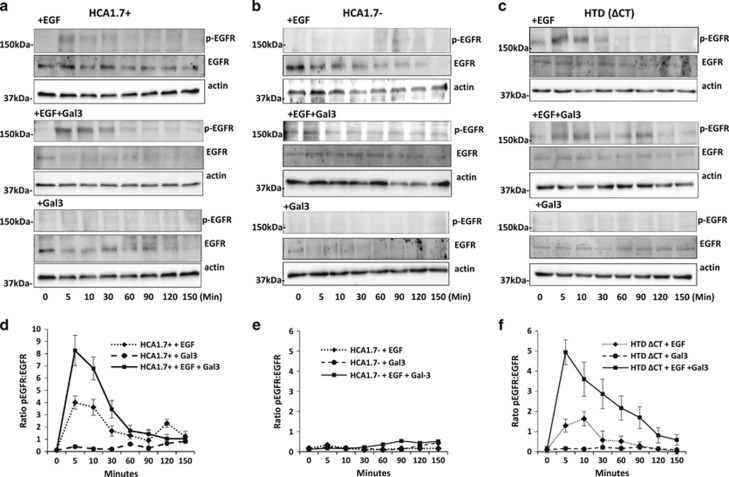
Galectin-3–MUC1 interaction promotes EGRF activation in human breast epithelial cells. HCA1.7+ (**a**), HCA1.7− (**b**) and HTD(ΔCT) (**c**) cells were treated with EGF in the presence or absence of galectin-3 for various time before analysed by immunoblotting with antibodies against p-EGFR, EGFR and actin. Galectin-3 treatment increased EGFR activation of the full-length MUC1 transfectants HCA1.7+ and the MUC1 cytoplasmic domain-depleted transfectants HTD(ΔCT), but not of the MUC1-negative revertants HCA1.7. Densitometry scanning of the bands from three independent experiments is shown in (**d**–**f**) and is expressed as ratio p-EGFR/EGF (mean±S.E.M.). Representative blots are shown in (**a**–**c**)

**Figure 4:**
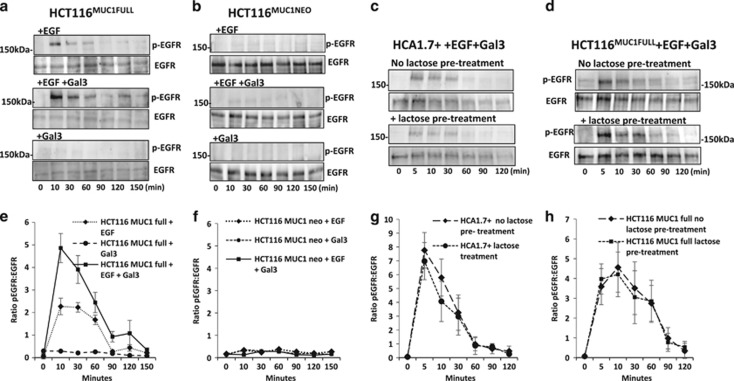
Galectin-3–MUC1 interaction enhances EGRF activation in human colon cancer cells. MUC1-expressing HCT116^MUC1Full^ (**a**) and MUC1-negative HCT116^MUC1neo^ (**b**) transfectants were treated with EGF in the absence or presence of galectin-3 for various time before analysed by immunoblotting with antibodies against p-EGFR, EGFR and actin. Galectin-3 treatment increased EGFR activation only in the MUC1-expressing but not MUC1-negative cells. In (**c**) and (**d**), HCA1.7+ and HCT116^MUC1Full^ cells were pre-treated with 100 mM lactose or PBS before introduction of EGF 20 ng/ml and 2 *μ*g/ml galectin-3 for various time and subsequent analysis of EGFR phosphorylation and EGFR expression by immunoblotting. Densitometry scanning of the bands from three independent experiments is shown in (**e**–**h**) and is expressed as ratio p-EGFR/EGF (mean±S.E.M.). Representative blots are shown in (**a**–**d**)

We next assessed the influence on EGFR activity of MUC1 cell surface interaction with galectin-3 at pathological galectin-3 concentrations observed in cancer patients.^24, 31^ Without the presence of EGF, introduction of galectin-3 had no significant effect on EGFR activation in either MUC1-positive or -negative cells of breast origin (Figures 3a–f). When EGF was introduced, galectin-3 presence caused more (e.g. by 106% at 5 min) EGFR activation in the MUC1-positive HCA1.7+ cells (Figures 3a and d) but had no effect in the MUC1-negative HCA1.7− cells (Figures 3b and e). In comparison to the cells treated with EGF alone, the presence of galectin-3 caused more (e.g. by 281% at 5 min) and prolonged EGFR activation in the MUC1-cytoplasmic domain-depleted HTD(ΔCT) cells (Figures 3c and f). Similar results were observed with colon cancer HCT116 cells (Figures 4a–d). Thus interaction of cell surface MUC1 with galectin-3 promotes EGFR activation.

We also assessed the contribution of endogenous galectin-3 to this cell surface interaction on EGFR activation. A previous study showed that treatment of HCT116 cells with 30 mM lactose could by competitive binding remove cell surface galectin-3.^32^ Using this strategy, we found little difference in EGFR activation between lactose pre-treated and untreated cells in both HCA1.7+ (Figures 4c and g) and HCT116^MUC1Full^ cells (Figures 4d and h) in response to EGF and galectin-3. This indicates that in this experimental setting the contribution of endogenous cell surface galectin-3 is minimal.

### EGFR activation induced by MUC1–galectin-3 interaction increases ERK1/2 signalling

It is known that EGFR activation on the cell membrane triggers an array of intracellular signalling pathways including ERK.^17, 33^

**Figure 5:**
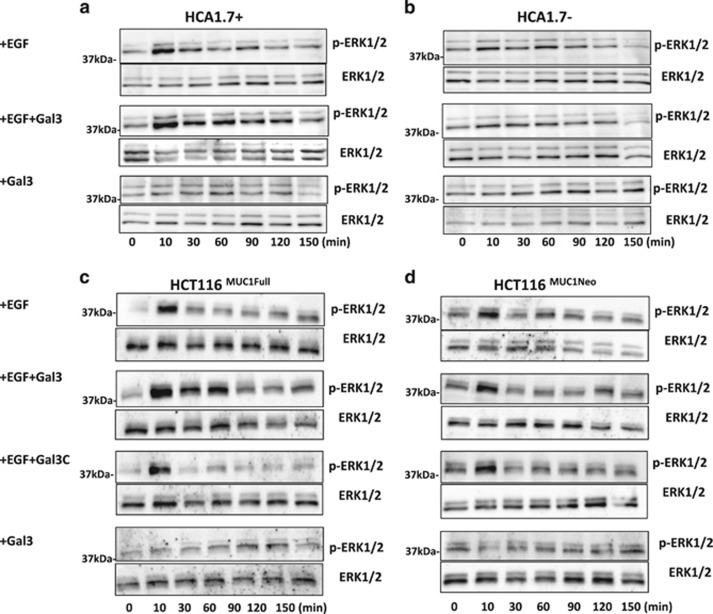
MUC1 expression- as well as MUC1–galectin-3 interaction-associated EGFR activation increases ERK activation. MUC1-expressing HCA1.7+ (**a**) and HCT116^MUC1Full^ (**c**), and MUC1-negative HCA1.7**−** (**b**) and HCT116^MUC1neo^ (**d**) cells were treated with either 20 ng/ml EGF, 20 ng/ml EGF and 2 *μ*g/ml galectin-3, 2 *μ*g/ml galectin-3 or 2 *μ*g/ml galectin-3C for various times as in Figures 3 and 4 before the expression of p-ERK1/2 and ERK1/2 were analysed by immunoblotting. EGF treatment increases ERK1/2 phosphorylation in the MUC1-expressing HCA1.7+ and HCT116^MUC1Full^ cells. Introduction of galectin-3, but not galectin-3C, further enhances ERK1/2 activation in the MUC1-expressing cells but not in the MUC1-negative cells. Representative blots from three independent experiments are shown

Introduction of EGF induced rapid ERK1/2 phosphorylation in MUC1-positive HCA1.7+ and HCT116^MUC1Full^ cells (Figures 5a and c). ERK1/2 phosphorylation peaked at 10 min with a 3.6- and 10.3-fold increase observed in HCA1.7+ (Figures 5a) and HCT116^MUC1Full^ (Figure 5c) cells respectively. Introduction of EGF also induced ERK1/2 phosphorylation of the MUC1-negative HCA1.7− and HCT116^MUC1neo^ cells but to a much lower level (Figures 5b and d), in consistence with the effect of MUC1 expression on EGFR activity shown in Figures 2,3,4. At 10 min, a 1.9- and 1.8-fold increase of ERK1/2 phosphorylation was observed in HCA1.7− and HCT116^MUC1neo^ cells.

When galectin-3 was also present, EGF induced a stronger (1.9- and 4.9-fold further increase at 10 min) and more prolonged ERK1/2 phosphorylation in the MUC1-positive HCA1.7+ (Figure 5a) and HCT116^MUC1Full^ (Figure 5c) cells, while ERK1/2 phosphorylation in the MUC1-negative HCA1.7− (Figure 5b) and HCT116^MUC1neo^ cells (Figure 5d) was unchanged. In contrast to the enhanced ERK1/2 activation by the full-length galectin-3/EGF in the MUC1-positive cells, introduction of C-terminal galectin-3 form (galectin-3C) with EGF showed no further effect on ERK-1/2 phosphorylation in comparison to treatment with EGF. Galectin-3 alone without EGF did not affect ERK1/2 phosphorylation.

These results suggest that, as predicted, EGFR activation induced by MUC1–galectin-3 interaction enhances and prolongs downstream ERK1/2 activation. The lack of effect by galectin-3C, in which its N-terminal ligand multimerization domain is depleted, suggests that MUC1 clustering is essential in galectin-3–MUC1 interaction-induced EGFR activation.

### Activation of EGFR and ERK by galectin-3–MUC1 interaction is inhibited by the EGFR inhibitor lapatinib

**Figure 6:**
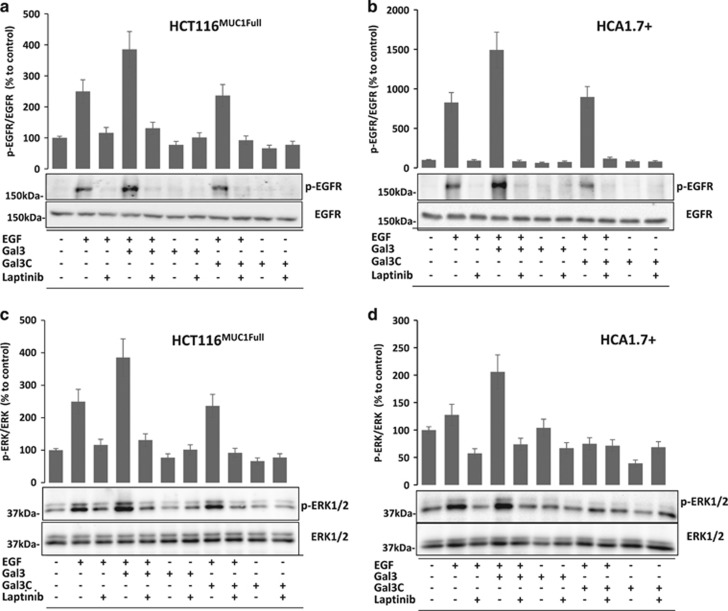
Lapatinib inhibits EGFR and ERK activation induced by MUC1–galectin-3 interaction. HCT116^MUC1Full^ (**a** and **c**) and HCA1.7+ (**b** and **d**) cells were treated with and without EGF in the absence or presence of galectin-3, galectin-3C, EGFR inhibitor lapatinib for 10 min before analysed by immunoblotting with antibodies against p-EGFR, EGFR (**a** and **b**) or pERK1/2 and ERK1/2 (**c** and **d**). Densitometry analysis of the bands from two independent experiments was quantified and was presented as percentage changes of p-EGFR/EGF and p-ERK1/2/ERK1/2, respectively, in comparison to the controls

To further determine whether the effect of galectin-3–MUC1 interaction on ERK activation was indeed the consequence of EGFR activation, we tested the effect of Lapatinib, an EGFR phosphorylation inhibitor^34^ on activation of EGFR and ERK in these cells. Lapatinib inhibited phosphorylation of EGFR and ERK1/2 in response to EGF and EGF plus galectin-3 in HCT116^MUC1Full^ (Figures 6a and c) and HCA1.7+ cells (Figures 6b and d). As above, the presence of galectin-3C did not affect phosphorylation of EGFR (Figures 6a and b) or ERK1/2 (Figures 6c and d). These results suggest that the increased phosphorylation of ERK1/2 by MUC1 and by MUC1–galectin-3 interaction (Figure 5) is the consequence of EGFR activation.

### Galectin-3–MUC1 interaction increases EGFR homo-/hetero-dimerization

In EGF-induced EGFR activation, EGFR dimerization occurs followed by EGFR auto-phosphorylation and internalization.^35, 36, 37^ We speculated that the effect of galectin-3–MUC1 interaction on EGFR activation might be associated with an effect on EGFR dimerization. To test this, we treated the cells without or with EGF or galectin-3 and then with non-cleavable crosslinker BS3 before EGFR analysis by immunoblotting.

**Figure 7:**
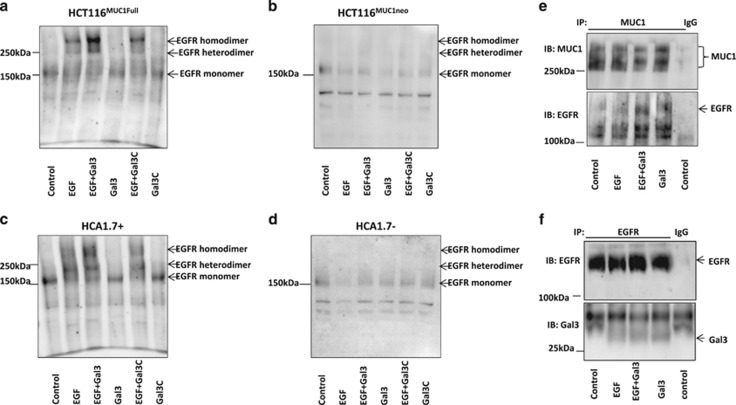
Galectin-3–MUC1 interaction promotes EGFR dimerization and MUC1–EGFR interaction. HCT116 ^MUC1Full^ (**a**), HCA1.7+ (**c**), HCT116^MUC1 neo^ (**b**) and HCA1.7− (**d**) were treated with and without EGF in the absence or presence of galectin-3 or galectin-3C for 10 min before EGFR dimerization were analysed using BS3 cross linker and immunoblotting. The presence of galectin-3, but not galectin-3C, increased EGFR homo- and hetero-dimerization in the MUC1-expressing, but not MUC1-negative, cells. HCA1.7+ (**e**) or HCT116^MUC1Full^ (**f**) cells were treated with PBS (control), EGF with or without galectin-3 for 10 min followed by immunoprecipitation of the cells with B27.29 anti-MUC1 antibody (**e**) or anti-EGFR antibody (**f**). The immunoprecipitates were analysed by immunoblotting with anti-EGFR, anti-MUC1 (B27.29) or anti-galectin-3 antibody. More EGFR was co-immunoprecipitated with MUC1 in cells treated with galectin-3 regardless of the presence of EGF (**e**). No difference of galectin-3 levels in the EGFR immunoprecipitates between cells treated with EGF and EGF plus galectin-3 (**f**)

It was found that, as expected, treatment of the cells with EGF induced EGFR dimerization in the MUC1-positive HCT116^MUC1Full^ (Figure 7a) and HCA1.7+ (Figure 7c) cells. EGFR dimerization was seen predominately as homo-dimers in HCT116^MUC1Full^ but hetero-dimers in HCA1.7+ cells in response to EGF. The presence of galectin-3 further increased EGFR dimerization in both cell types. Interestingly, galectin-3-induced EGFR dimerization involved both homo- and hetero-dimers in HCT116^MUC1Full^ cells (Figure 7a) but predominately homo-dimers in HCA1.7+ cells (Figure 7c). EGF alone, or with galectin-3, showed little effect on EGFR dimerization in the HCT116^MUC1neo^ (Figure 7b) and HCA1.7− (Figure 7d) cells. Moreover, although the presence of full-length galectin-3 increased EGFR dimerization (Figures 7a and b) and EGFR phosphorylation (Figures 3–6), the truncated galectin-3C did not show any effect on EGFR dimerization.

These results suggest that EGFR activation induced by galectin-3–MUC1 interaction is associated with promotion of EGFR dimerization. The lack of effect of the truncated galectin-3C on EGFR dimerization further supports an important role of galectin-3-induced MUC1 clustering in EGFR activation.

### Galectin-3 increases interaction of MUC1 with EGFR

We also assessed interaction of MUC1 with EGFR in cell response to EGF and galectin-3. It was found treatment of the cells with EGF did not have any effect on MUC1–EGFR interaction (Figure 7e). However, treatment of the cells with galectin-3, regardless of the presence or absence of EGF, resulted in increased co-immunoprecipitation of EGFR with MUC1 (Figure 7e). This suggests that galectin-3–MUC1 interaction promotes physical interaction of MUC1 with EGFR and this likely represents a key component of galectin-3-associated EGFR activation.

Galectin-3 has been previously reported to be able to interact directly with EGFR^16, 38^; however, we found minimal galectin-3 co-immunoprecipitation with EGFR (Figure 7f). In comparison to EGF alone-treated cells, introduction of galectin-3 and EGF did not increase galectin-3 presence in EGFR immunoprecipitates, thus not supporting a role of galectin-3–EGFR interaction in this action of EGFR activation.

### Galectin-3 increases EGFR internalization

**Figure 8:**
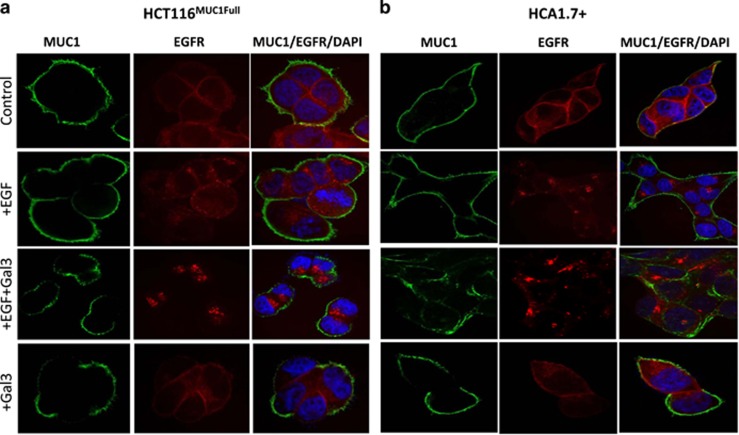
Galectin-3–MUC1 interaction enhances EGFR internalization. HCT116^MUC1Full^ (**a**) and HCA1.7+ (**b**) cells were treated with PBS (control), EGF with or without galectin-3 for 10 min before localization of MUC1 (green) and EGFR (red) were determined by fluorescent immunohistochemistry and analysed by confocal microscopy. The cell nucleus was stained with DAPI (blue). Galectin-3 changes MUC1 cell surface localization (as illustrated by disruption of uniform MUC1 localization). More intense and clustered EGFR localization inside the cells were seen in the galectin-3 treated cells than in the EGF alone treated cells in both cell types. Representative images are shown

Following EGFR ligand binding, dimerization and auto-phosphorylation, EGFR internalization is an essential next step in EGFR signalling. In both HCT116^MUC1Full^ and HCA1.7 cells, EGFR appeared both on the cell surface and inside the cells (Figures 8a and b). Addition of EGF resulted in substantial loss of EGFR from the cell surface and its intra-cellular accumulation in both HCT116^MUC1Full^ (Figure 8a) and HCA1.7+ (Figure 8b) cells. MUC1 was uniformly spread on the cell surface and unaffected by the absence or presence of EGF. Introduction of galectin-3, as reported previously,^11^ disrupted the uniform MUC1 cell surface localization. The presence of galectin-3 with EGF also increased EGFR internalization in comparison to the cells treated with EGF alone and caused a more clustered pattern of internalized EGFR. Introduction of galectin-3 without EGF did not affect EGFR localization. This, together with the lack of effect of full-length galectin-3 on EGFR activation in MUC1-negative cells and the lack of effect of truncated galectin-3C on EGFR activation in the MUC1-positive cells, indicates that galectin-3-mediated EGFR activation is associated with its effect on alteration of MUC1 cell surface localization.

## Discussion

This study shows that EGF-induced EGFR activation is substantially increased by expression of MUC1 in human breast and colon epithelial cells. Both the MUC1 intracellular and extracellular domains contribute to the effect of MUC1 on EGFR activation but the predominant influence comes from the MUC1 extracellular domain. Interaction of cell surface MUC1 with galectin-3 induces changes of MUC1 cell surface localization and increases MUC1–EGFR interaction. This leads to an increase of EGFR homo-/hetero-dimerization and subsequently increased EGFR activation and signalling. This effect of galectin-3 occurs only with the full length but not the truncated galectin-3 form that lacks its N-terminal domain responsible for galectin-3-mediated receptor clustering.

Over-expression of MUC1 is common in cancer^39^ and it is associated with EGFR in epithelial cancers including breast,^28, 40^ pancreatic,^16^ endometrial^14^ and lung.^41^ Blocking MUC1-C terminal dimerization with a cell-penetrating peptide^42^ or siRNA silencing MUC1-C expression^43^ has been shown to suppress EGFR activation-associated cell signalling and survival in non-small cell lung cancer cells. Interaction of MUC1 with EGFR in the nucleus of breast cancer cells promotes accumulation of chromatin-bound EGFR and co-localization of EGFR with phosphorylated RNA polymerase II.^28^ The present study shows that MUC1 expression increases EGF-induced EGFR activation in human breast and colon cancer cells. Depletion of either the MUC1 intracellular or extracellular domain could only partly abolish MUC1-associated effect on EGFR activation. This is in keeping with previous studies showing that the MUC1 extracellular^42^ and cytoplasmic^43^ domains can both interact with EGFR and affect EGFR activity although here we show that the predominate influence of MUC1 on EGFR activation derives from its extracellular domain. MUC1^13^ and EGFR^44^ have both been shown to be associated with lipid rafts on the cell membrane. It seems likely that their interaction within the lipid raft may facilitate the formation of EGFR homo-/hetero-dimers in response to ligand banding.

Interaction between MUC1 and full-length galectin-3 is known to induce MUC1 cell surface polarization.^11, 26, 27^ The effect of galectin-3 on MUC1 cell surface localization was indeed visible in this study, irrespective of the presence or absence of EGF (Figure 8). However, galectin-3 enhances EGFR activation only when EGF is also present (Figures 5 and 6). This indicates that galectin-3 cannot activate EGFR without the presence of an EGFR ligand. MUC1 cell surface polarization induced by MUC1–galectin-3 interaction has been shown previously to expose underlying smaller cell surface molecules.^11, 26, 27^ However, the discovery that EGF showed a much weaker effect on EGFR activation in the MUC1-negative cells (Figures 2–5), irrespective of the presence of galectin-3, indicates that exposure of cell surface EGFR for easy EGF access is unlikely to be the mechanism of the MUC1–galectin-3 interaction-associated EGFR activation.

MUC1 co-immunoprecipitation showed a weak presence of EGFR in MUC1 immunoprecipitates but a substantial increase after addition of galectin-3, with or without the presence of EGF (Figure 7e). This, together with the discovery that galectin-3 alone did not induce EGFR dimerization, suggests that galectin-3–MUC1 interaction is essential for galectin-3-associated, EGF-induced EGFR activation. The importance of galectin-3-mediated change of MUC1 cell surface localization in EGFR activation is supported by the evidence that truncated galectin-3C lacking the N-terminal domain responsible for galectin-3-induced ligand clustering could not induce MUC1 polarization, and did not affect EGFR dimerization or downstream signalling (Figures 5–7).

An earlier study suggested that galectin-3 might form a bridge between MUC1 and EGFR in cancer cells.^43^ In our study, very minimal galectin-3 was co-immunoprecipitated with EGFR and addition of exogenous galectin-3 also showed no effect on EGFR association with galectin-3 (Figure 7f). Addition of galectin-3 alone also did not show any effect on EGFR phosphorylation, EGFR dimerization or ERK activation in the MUC1-positive cells in the absence of EGF, nor did it show any effect on EGFR activation in the MUC1-negative cells even in the presence of EGF (Figures 2–7). This indicates that a direct binding of galectin-3 to EGFR, even if it occurs, does not contribute to galectin-3–MUC1-associated EGFR dimerization and activation in these cells. Another study has reported a role of galectin-3 in promoting spheroid formation of lung cancer cells through activation of EGFR.^38^ Although that study did not identify the relevant galectin-3 binding ligand, their discovery of the requirement of the galectin-3 carbohydrate recognition domain is broadly in keeping with the effect of galectin-3–MUC1 interaction on EGFR activation, as shown here.

It was found here that the contribution of endogenous cell surface galectin-3 to EGFR activation is minimal. Our previous study has shown that galectin-3 secretion in these cells in such a short-term cell culture condition is minimal.^27^ In cancers, galectin-3 is typically constantly secreted and could reach higher levels which would impact on EGFR activation by interaction with cancer-associated MUC1. Moreover, the concentration of exogenous galectin-3 used in this study is close to the pathological level of circulating galectin-3 in metastatic cancer patients.^24^ The impact of exogenous galectin-3 on EGFR activation via interaction with MUC1 reported in this study is therefore likely to be particularly relevant to circulating tumour cells during metastasis.

Galectin-3–MUC1 interaction causes a prolonged activation of EGFR and ERK (Figure 5). It is generally believed that EGFR activation is terminated primarily through endocytosis of the receptor–ligand complex which is either degraded in the endosomes or recycled to the cell surface. It has been reported that if recycled EGFR is unable to reach the cell surface or to the lysosomal compartment but accumulates in the early endosomes, it will lead to prolonged signalling and increased activation of ERK.^45^ This does seem to be supported in our study. We found that following EGFR activation, more EGFR was seen to be located in a clustered pattern inside the cells in response to galectin-3/EGF treatment compared to EGF alone (Figure 8). There was also a much weaker EGFR cell surface localization in the galectin-3/EGF treated cells suggesting that the galectin-3/MUC1-mediated EGFR activation and subsequent EGFR endocytosis is associated with slower recycling of EGFR to the cell surface (Figure 5). This is in keeping with an earlier study showing that MUC1 expression inhibits EGFR degradation in response to ligand binding but was accompanied by an increase of EGFR internalization in breast epithelial cells.^46^

The EGFR phosphorylation inhibitor lapatinib completely inhibited EGFR phosphorylation (Figures 6a and b) but some ERK activity remained in the cells even in the absence of EGF (Figures 6c and d). ERK is known to be regulated by a variety of growth factors and molecules^47^ and expression for example of either galectin-3^16, 32, 48^ or MUC1^49^ in cancer cells has previously been shown to induce ERK activation.

EGFR represents a key therapeutic target for cancer treatment. The demonstration here that the expression of MUC1 and its interaction with galectin-3 promotes ligand-dependent EGFR activation has implications in EGFR-targeted therapies. MUC1 and galectin-3 are both commonly over-expressed by solid tumours and their interactive effects on EGFR activation may have an important influence on EGFR-mediated tumourigenesis and cancer progression, and on the effectiveness of EGFR-targeted therapy. For example, a closer localization of EGFR with MUC1 on the cell membrane induced by galectin-3–MUC1 interaction may limit the access of anti-EGFR antibodies to cell surface EGFR due to the massive size of MUC1 that easily protrudes over EGFR on the cell surface. A slower recycling of EGFR to the cell surface induced by galectin-3–MUC1 interaction may also limit the treatment effectiveness of anti-EGFR antibody as well as kinase inhibitors. It is possible therefore that a combined therapy that targets EGFR as well as galectin-3 or MUC1 may improve treatment effectiveness in patients who have increased expressions of galectin-3 and MUC1.

## Materials and methods

Antibodies against p-EGFR (SC-23420) EGFR (SC-03), p-ERK1/2(SC-7383) and ERK1/2 (SC-94), Protein A/G plus agarose beads were purchased from Santa Cruz Biotechnology (Santa Cruz, CA, USA). Anti-EGFR antibody used in confocal microscopy and for immunoprecipitation (DB81) was from New England Bio-Labs (Ipswich, MA, USA). Anti-EGFR antibody (500-p306) and recombinant human EGF (AF-100-15) was from PeproTech (Rocky Hill, NJ, USA). Bis(sulfosuccinimidyl) substrate (BS3) crosslinker was purchased from Life Technology Ltd (Waltham, MA, USA). Lapatinib was purchased from Sigma-Aldrich (Dorset, UK). B27.29 anti-MUC1 antibody was kindly provided by Dr. Mark Reddish (Biomira, Edmonton, Canada) and CT2 anti-MUC1 antibody were kindly provided by Prof. Sandra Gendler (Mayo Clinic, Scottsdale, AZ, USA).

### Cell lines

Human colon cancer HCT116 cells were obtained from European Collection of Cell Cultures (Salisbury, UK) and were cultured in McCoy’s 5a medium. MUC1-positive transfectants (HCA1.7+), MUC1 negative revertants (HCA1.7−) and MUC1 cytoplasmic domain-depleted MUC1 mutant (HTDΔCT) from human breast HBL-100 epithelial cells were described previously.^5^ The cell lines were last authenticated by DNA profiling (DNA Diagnostics Centre, London, UK) in 2014.

### MUC1 transfection

MUC1 expression vectors for full-length MUC1, the extracellular domain-depleted MUC1 (MUC1ΔTR), the cytoplasmic domain-depleted MUC1 (MUC1ΔCT) and control vector were kindly provided by Prof. Tony Hollingsworth (University of Nebraska Medical Centre). MUC1-expressing or control vector was pre-mixed with DNA Diluent and hydrated GenePOORTER-2 transfection reagent in serum-free medium for 10 min before addition to HCT116 cells in antibiotics-free and serum-containing DMEM in 24-well plates for 24 h at 37 °C. The culture medium was replaced with serum-containing medium for 48 h before the cells were cultured in normal medium containing 600 *μ*g/ml G418 for 7–10 days at 37 °C. Single-cell clones were selected with Cell Cloning Cylinders, proliferated and analysed for MUC1 expression by immunoblotting with anti-MUC1 antibodies B27.29 and CT2.

### Production of full-length and truncated forms of recombinant galectin-3

The cDNA sequence encoding full-length human galectin-3 (Gal-3F) and C-terminal carbohydrate recognition domain (CRD) of galectin-3 (Gal-3C) (residues 116–250) were cloned into pETm11 expression vector with a His-tag. The recombinant plasmids were transformed into Bl21(DE3) *Escherichia coli* and the transformants were selected with kanamycin. The protein expression was induced using 1 mM IPTG when the cell density (OD_600_) reached approximately 0.6–0.85. Following induction, cells were incubated overnight at 18 °C before harvested by centrifugation. The cells were lysed in the presence of DNase using high pressure cell homogeniser. After centrifugation, the supernatant was applied onto a HisTrap FF 5 ml column (GE Healthcare, Buckinghamshire, UK) and the His-tagged proteins were eluted with 150 mM Imidazole. The collected fractions containing galectin-3 were incubated overnight with TEV protease to cleave the His tag and dialysed against His Trap buffer without Imidazole. After performing Reverse His Trap to remove the cleaved His tag and TEV protease from galectin-3 solution, the proteins were further purified by size exclusion chromatography using Superdex 75 26/60 column. The purified Gal-3C was eluted between 220 and 260 ml and the Gal-3 F between 190 and 220 ml. Purify of the recombinant proteins was determined by SDS–PAGE to be >95%.

### Immunoprecipitation

Sub-confluent cells were incubated in serum-free medium containing 0.5 mg/ml BSA overnight. The cells were washed with TBS and then incubated with EGF (20 ng/ml), or EGF (20 ng/ml) and galectin-3 (2 *μ*g/ml), or galectin-3 (2 *μ*g/ml) or 20 ng/ml BSA (control) in serum-free media for 10 min at 37 °C. The cells were washed with ice-cold PBS, scraped and lysed on ice in PBS containing 1% Triton-X-100 and protease inhibitors (Calbiochem, Hertfordshire, UK) for 30 min before centrifugation at 10 000 × *g* at 4 ^°^C for 15 min. The supernatants were collected and pre-cleared by adding 20 *μ*l of the protein-A/G beads and incubating at 4 °C for 30 min with gentle agitation. One millilitre lysates (protein concentration 2 mg/ml) were incubated with anti-MUC1 (B27.29, 1 *μ*g/ml), anti-EGFR (DB81) (2 *μ*g/ml) antibody or isotype-matched normal IgG at 4 °C with continuous agitation for 16 h. Thirty *μ*l of protein- A/G plus agarose beads were added for 4 h and the beads were washed five times with ice-cold PBS. Proteins were eluted from the beads by boiling in SDS-sample buffer for 10 min before application to SDS–PAGE and subsequent immunoblotting

### Immunoblotting

Cellular proteins (cell lysate or immunoprecipitates) separated by SDS–PAGE were electro-transferred to nitrocellulose membrane. The membranes were first incubated with specific primary antibodies (anti-p-EGFR (SC-23420), EGFR (SC-03), anti-pERK (SC-7383)) and ERK (SC-94) at a concentration of 1:500. Antibodies against MUC1 (B27.29, CT2) or actin at a concentration of 1:5000 were applied for 16 h at 4 ^°^C. The blots were washed three times with 0.05% Tween-20 in TBS before incubated with peroxidase-conjugated secondary antibody (1: 3000) for 1 h. After six washes with 0.05% Tween-20 in TBS, the protein bands were developed using chemiluminescence Super Signal kit and visualized with Molecular Imager Gel Doc XR System (Bio-Rad, Hempstead, UK). The density of the protein bands was quantified using Imagelab version 3.0.1.

### EGFR activation

Sub-confluent cells were incubated in serum-free medium containing 0.5 mg/ml BSA overnight. The cells were washed with PBS before incubation with EGF (20 ng/ml), EGF (20 ng/ml) and galectin-3 (2 *μ*g/ml), galectin-3 (2 *μ*g/ml), galectin-3C (2 *μ*g/ml) or BSA (2 *μ*g/ml) (control) in the absence or presence of EGFR inhibitor lapatinib (2 mM) for various time at 37 ^°^C and 5% CO_2_. In some experiments, the cells were first incubated with 100 mM lactose or PBS for 30 min before washing and application of EGF or EGF plus galectin-3 for various time at 37 °C. The cells were washed immediately with ice-cold TBS before lysed with SDS-sample buffer and analysed by immunoblotting.

### Cell surface protein crosslinking

Sub-confluent cells were incubated in serum-free medium overnight. The cells were washed twice with Ca^2+^- and MG^2+^-free PBS and then treated with serum-free media containing BSA 2 *μ*g/ml (control), EGF (20 ng/ml) without or with galectin-3 (2 *μ*g/ml) or galectin-3C (2 *μ*g/ml) for 10 min at 37 °C and 5% CO_2_. The cells were then washed with ice-cold Ca^2+^- and MG^2+^-free PBS and incubated with 3 mM BS3 crosslinker in Ca^2+^- and MG^2+^-free PBS on ice for 20 min. Excess BS3 was quenched with 250 mM glycine in PBS for 5 min at 4 °C. The cells were washed three times with ice-cold PBS, lysed in SDS-sample buffer and analysed by immunoblotting with antibodies against EGFR.

### Confocal microscopy

Sub-confluent cells grown on glass coverslips in 24-well plates were incubated in serum-free at 37 °C overnight. The cells were treated with BSA (2 *μ*g/ml) (control), EGF (20 ng/ml) without or with galectin-3 (2 *μ*g/ml) for 10 min at 37 ^°^C. The cells were washed with ice-cold PBS and fixed with 4% paraformaldehyde. The cells were then washed again with PBS and probed with anti-MUC1 B27.29 (1 *μ*g/ml) or anti-EGFR (D38B1) (2 *μ*g/ml) for 2 h at room temperature. After two washes with PBS, FITC-conjugated anti-mouse or Alexa fluor 643-conjugated anti-rabbit antibodies were applied for 1 h at room temperature. The cells were washed twice before being mounted using DAPI-containing fluorescent mounting media (Vector Laboratories, Burlingame, CA, USA). The slides were analysed using a 3i confocal microscope (Marianas SDC, 3i Imaging) and Slidebook 6 Reader version 6.0.4 (Intelligent-imaging).
